# Time-Restricted Eating and Symptom Severity in Irritable Bowel Syndrome: Results from a Pilot Study

**DOI:** 10.3390/nu18050765

**Published:** 2026-02-26

**Authors:** Maria Thompson Clausen, Henrik Sverdrup, Asgeir Brevik, Marianne Molin, Marit Kolby

**Affiliations:** 1 Department of Health and Exercise, School of Health Sciences, Kristiania University College of Applied Sciences, P.O. Box 1190, N-0107 Oslo, Norway; mthclausen@gmail.com (M.T.C.); henrik@baumann.no (H.S.); marianne.molin@kristiania.no (M.M.); 2 Institute of Basic Medical Sciences, University of Oslo, P.O. Box 1110, N-0317 Oslo, Norway; 3 Department of Nursing and Health Promotion, Faculty of Health Sciences, Oslo Metropolitan University, P.O. Box 4, N-0130 Oslo, Norway; asgeir@oslomet.no; 4 Department of Health, Oslo New University College, Ullevålsveien 76, N-0454 Oslo, Norway

**Keywords:** time-restricted eating, irritable bowel syndrome, meal timing, dietary intervention, intermittent fasting

## Abstract

Background/Objectives: Irritable bowel syndrome (IBS) is a functional gastrointestinal disorder affecting approximately 5–10% of the population, with many individuals reporting insufficient improvement from treatment options. This study aimed to explore whether time-restricted eating (TRE) could alleviate symptoms in patients with IBS. Methods: This single-group, pre–post pilot study included participants with IBS who followed an 8-week time-restricted eating protocol, defined as a daily 16 h fasting period and an 8 h eating window (16:8). Symptom changes were assessed using the validated IBS Symptom Severity Scale (IBS-SSS) at baseline and post-intervention. The study was retrospectively registered after completion of data collection. Results: A total of 134 patients were enrolled, of whom 97 completed the intervention. Participants demonstrated a mean reduction in the IBS-SSS score of −100.2 (*p* < 0.001). Subgroup analysis also demonstrated mean reductions in the IBS-SSS scores for participants with IBS-constipation (IBS-C) (−125.2, *p* < 0.001), IBS-diarrhea (IBS-D) (−76.0, *p* < 0.005), and IBS-mixed (IBS-M) (−93.1, *p* < 0.001). Additionally, the participants experienced improvements in both self-reported physical and mental health. Conclusions: These preliminary findings suggest that TRE may represent a promising behavioral strategy for IBS symptom management, warranting controlled trials.

## 1. Introduction

Irritable bowel syndrome (IBS) is a common functional gastrointestinal disorder, characterized by recurrent abdominal pain associated with altered bowel habits [1]. Diagnosis is most often established using the Rome IV criteria, which relies on the evaluation of symptom patterns in the absence of detectable structural abnormalities [2]. The different subtypes of IBS, including IBS with constipation (IBS-C), with diarrhea (IBS-D), IBS mixed (IBS-M), and IBS unclassified (IBS-U), are defined by the predominant stool morphology during abnormal bowel movement. The global prevalence of irritable bowel syndrome is estimated to range from 4% to 10%, with variations depending largely on the diagnostic criteria applied [3]. IBS disproportionately affects women and younger adults, although men are also substantially affected [3,4,5]. Living with IBS can present significant challenges. Pain, bowel difficulties and bloating contribute to the severity of the condition, whereas dietary restrictions to manage symptoms add to the burden of the condition [6]. IBS is associated with substantial reductions in quality of life and represents a significant psychosocial and economic burden for many patients [7,8,9].

### 1.1. Pathophysiology

The pathophysiology of IBS is multifactorial and incompletely understood. Proposed mechanisms include altered gut–brain signaling, visceral hypersensitivity, microbiota changes, and low-grade immune activation [10,11,12,13,14]. The heterogeneity of these mechanisms contributes to variable treatment responses and highlights the need for diverse therapeutic strategies.

### 1.2. Dietary Management of IBS

Dietary factors play an important role in symptom manifestation; therefore, dietary management is central to IBS treatment [14]. The specific types of food that trigger the symptoms and the nature of these symptoms can vary among individuals. Among the most well-known dietary triggers are specific carbohydrates such as fermentable oligosaccharides, disaccharides, monosaccharides, and polyols (FODMAPs), which appear to cause symptoms in IBS patients [3]. These carbohydrates are found in dairy products containing lactose, legumes, sugar alcohols, vegetables, nuts, cereals, and some fruits. FODMAPs generally pass through the small intestine to the colon without undergoing digestion [15]. The mechanism behind the intolerance to FODMAPs is not fully understood but could partly be explained by inter-individual differences in the ability to digest certain FODMAPs due to lack or reduced levels of necessary enzymes [15].

Current recommendations, such as the NICE guidelines and the low-FODMAP diet, may alleviate symptoms but can be restrictive and difficult to maintain over the long term. A very low-carbohydrate diet also appears to exert beneficial effects on symptom severity, particularly in individuals with IBS-D [16]. Adjuncts include selected fibers, probiotics, digestive enzymes, pharmacotherapies, psychological therapies, and investigational strategies such as fecal microbiota transplantation (FMT), however with mixed results [14,17]. Owing to the heterogeneous pathophysiology of IBS patients and their varying responses to current treatment options, there is a need to expand available therapeutic approaches.

### 1.3. The Rationale for Time-Restricted Eating in IBS

Time-restricted eating (TRE) is a behavioral eating pattern that limits daily food intake to a consistent time window without restricting food type or energy intake [18]. Compared with exclusion-based dietary regimens, TRE targets meal timing rather than dietary composition and may therefore represent a more flexible intervention.

Mechanistic studies suggest that fasting influences biological systems implicated in IBS, such as gastrointestinal motility, immune regulation, and microbiota dynamics [19,20,21,22,23,24,25,26,27,28]. These observations provide biological plausibility for investigating TRE in this population.

Despite extensive research on composition-based dietary interventions in IBS, the temporal structure of eating has received little attention. Current dietary strategies focus primarily on food exclusion or macronutrient manipulation, often resulting in restrictive regimens that are difficult to sustain. In contrast, TRE targets meal timing alone and represents a conceptually different approach. To date, no study has examined whether restricting the daily eating window influences IBS symptom severity. This pilot study aimed to explore whether an 8-week TRE intervention (16:8) protocol could reduce IBS-related symptoms.

## 2. Materials and Methods

**Figure 1:**
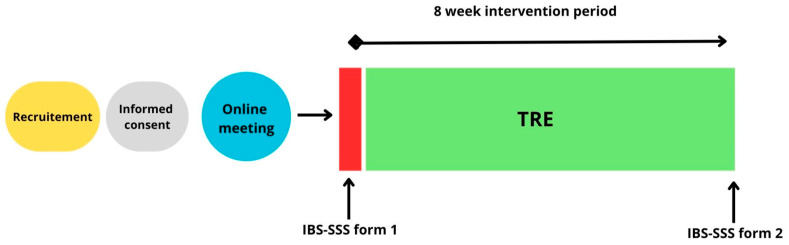
Design of the single group, pre-post intervention. Participants completed an 8-week TRE intervention. Symptom severity was assessed at baseline and after the intervention.

This single-group intervention pilot study examined the effects of TRE on symptom severity in patients with IBS. Participants completed an 8-week dietary intervention, with outcome measures assessed at baseline and post-intervention for the primary study period (Figure 1). An additional follow-up assessment was conducted at approximately 12 months. The 12-month follow-up was exploratory and analyzed separately from the primary 8-week outcomes. The study was not prospectively registered in an international clinical trial registry. To enhance transparency, the study was retrospectively registered in the Open Science Framework (OSF) after completion of data collection (registration date: 22 December 2025) [29].

### 2.1. Population and Recruitment

**Figure 2:**
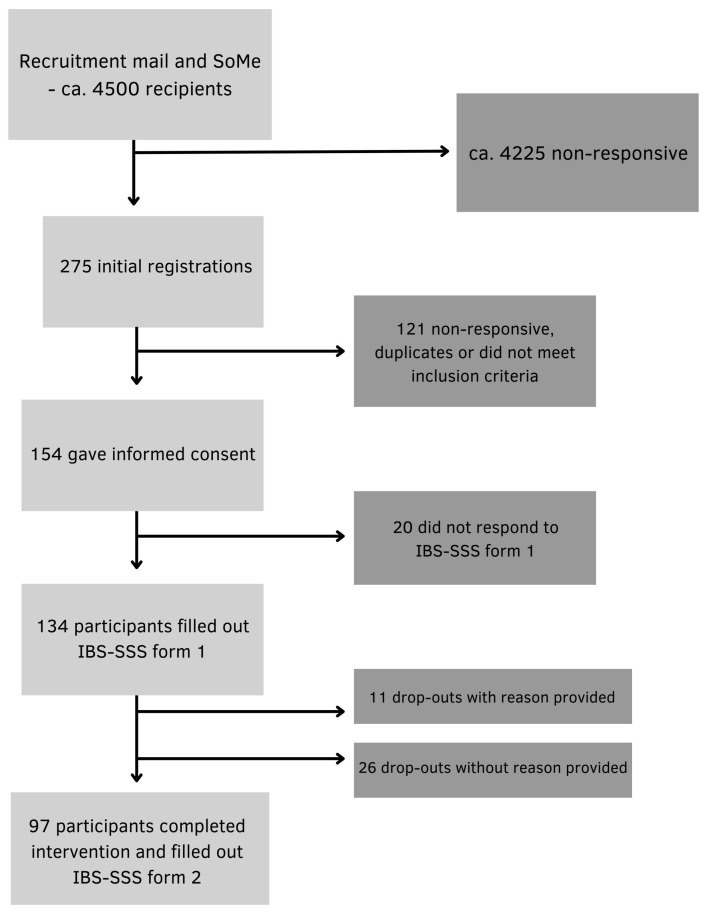
Flow chart of the recruitment of participants.

Participants were recruited from the Norwegian Gastrointestinal Association (approximately 4500 members), via information letters published on the association’s website, email and posts on social media platforms. Participants ≥ 18 years of age with a IBS diagnosis and no prior experience with intermittent fasting methods were included. IBS diagnosis was based on self-report of prior clinical diagnosis from a primary care physician or specialist. Diagnostic status was not independently verified by the research team, introducing a potential risk of misclassification bias. The exclusion criteria were pregnancy, breastfeeding, underweight (BMI < 18.5 kg/m^2^), history of surgery resulting in altered gastrointestinal anatomy, eating disorders and other conditions with symptoms similar to IBS. 134 participants provided digitally obtained informed consent and were enrolled in the study. A total of 97 participants completed the intervention and were included in the final analysis (Figure 2).

### 2.2. Intervention

The intervention was a pre/post design TRE regimen (16:8) where all participants were instructed to restrict their daily food intake to an 8 h window while fasting for 16 h between 16 September and 10 November 2024. One week prior to the intervention, two identical (to provide choice of participation times) digital information sessions were held, a private Facebook group was used for communication and support throughout the 8-week intervention. Participants were allowed to self-select an early or late eating window. These categories reflected individual preferences rather than fixed clock-based schedules; the instruction was to aim for 16 h of daily fasting in a way that was individually sustainable. Flexibility to switch between early and late schedules during the intervention period was permitted to enhance feasibility and adherence. During fasting, participants were instructed to consume only water and coffee/tea without milk and sweeteners. The use of sugar-free gum, lozenges, and snuff was discouraged but did not constitute an exclusion criterion. Within the eating window, participants were encouraged to eat to satiety without energy restrictions, and to consume fewer, larger meals to limit snacking. Maintenance of body weight was an explicit objective of the intervention and was emphasized throughout the study period. No objective adherence tracking (e.g., dietary logs or digital monitoring) was implemented. This should be considered when interpreting the results. 

### 2.3. Data Collection and Statistical Analyses

Data were collected using secure online questionnaires at baseline and post-intervention, administered via Nettskjema, a secure digital survey platform. The severity of symptoms was assessed using the IBS Symptom Severity Scale (IBS-SSS) from a validated Norwegian translation of the IBS-SSS, approved by the Rome Foundation [30], where questionnaire items are categorized into domains that assess pain, bloating, bowel habits, and quality of life, yielding a total IBS-SSS score ranging from 0 to 500. Within the range of the score, 75–175 is considered mild, 175–300 moderate and >300 severe, respectively. Participants also self-evaluated their physical and mental health according to 1–10 Likert scales. All questionnaire items were mandatory, except for body weight. No data imputation was performed. Analyses were conducted using complete cases only, including participants who completed both baseline and follow-up assessments (per-protocol analysis). Analyses were performed using STATA v18, StataCorp LLC, College Station, TX, USA. Continuous variables were assessed for normality using (Shapiro–Wilk test). Paired *t*-tests were applied to normally distributed data, and Wilcoxon signed-rank tests were used otherwise. Changes in the IBS-SSS were examined for the total sample and IBS subtype. Baseline characteristics are reported as mean ± SD or median (IQR). Statistical significance was set at *p* < 0.05.

## 3. Results

134 of the invited participants completed the pre-intervention questionnaire and were enrolled in the study. During the 8-week intervention, 37 participants discontinued participation: 11 with reasons provided and 26 without. A total of 97 participants completed the post intervention questionnaire and were included in the final analysis.

### 3.1. Participant Characteristics

Of the 97 participants, 90 (92.8%) were women and 7 (7.2%) were men (Table 1). The mean age was 42.5 (SD 12.9). Most of the participants had mixed IBS subtypes (42.3%), followed by constipation (29.9%), diarrhea (21.7%), and unclassified type (6.2%).

### 3.2. Results at 8 Weeks

**Figure 3:**
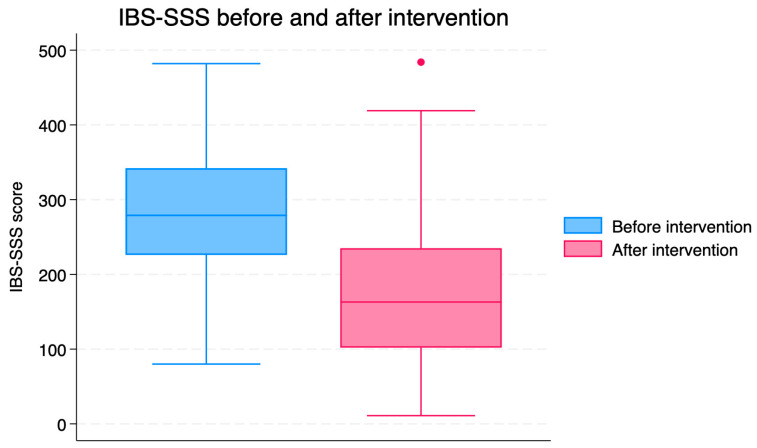
IBS-SSS scores before and after the intervention (n = 97). Each boxplot displays the median, interquartile, and full range of scores. The dot represents an outlier.

The total IBS-SSS score was reduced after 8 weeks of TRE (*p* < 0.001; Table 2, Figure 3). The mean change in the IBS-SSS score was −100.2 [SD 112.3]. Although substantial reductions in IBS-SSS scores were observed, the single-group pre–post design does not permit causal inference, and the findings should be interpreted as exploratory.

Subgroup analyses indicated that participants with IBS-C exhibited the most significant improvements, with a mean decrease in the total score of −125 (SD 117.9) (*p* < 0.001) for within-group pre–post change (Table 3), followed by participants with IBS-M and IBS-D, −93.1 (SD 107.4) (*p* < 0.001), and −76 (SD 100.4) (*p* < 0.005), respectively. In contrast, there was no statistically significant change in the small IBS-U group (n = 6).

Sixty participants (61.9%) exhibited a reduction of ≥50 points, indicating a clinically significant improvement [31]. Based on the total symptom scores, 12 individuals (12.4%) met the criteria for remission after eight weeks (Table 4). Post-intervention, 39 (40.2%) patients were classified within the mild severity range compared to 15 (15.5%) at baseline. The proportion of individuals with moderate severity decreased slightly from 44 (45.5%) before intervention to 36 (37.1%) after intervention. Notably, the number of participants with severe severity decreased from 38 (39.2%) at baseline to 10 (10.3%) following the intervention (Table 4).

**Figure 4:**
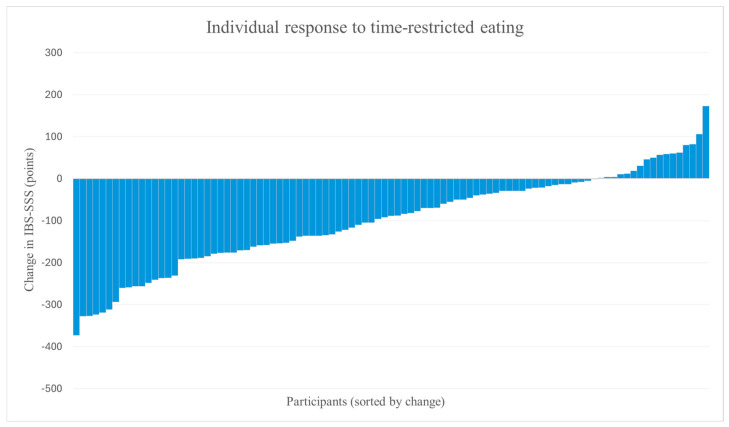
Individual changes in IBS-SSS scores from baseline to post-intervention, following time-restricted eating (n = 97). The waterfall plot displays individual participant changes in IBS-SSS, sorted by the magnitude of change. Negative values indicate improvement; positive values indicate worsening of symptoms.

There was considerable inter-individual variability in response to the intervention. While most participants demonstrated improvements, a smaller number of participants exhibited minimal change or worsening of symptoms (Figure 4).

Self-reported physical and mental health scores increased modestly but significantly from baseline to after intervention. Mean physical health improved from 6.2 (SD 1.6) to 6.7 (SD 1.6) and mean mental health from 6.7 (SD 2.0) to 7.0 (SD 1.7), corresponding to modest but statistically significant changes (*p* < 0.05 for both).

Body weight changed modestly during the intervention. Twenty-eight participants maintained stable weight, 20 experienced an increase in BMI, and 48 showed a reduction. The mean change in body weight across all participants was −0.71 kg.

### 3.3. Results at Follow-Up (12 Months)

At 12-month follow-up, 58 participants (59.8%) responded to a digital questionnaire assessing IBS symptoms, self-reported physical and mental health, and current practice of TRE.

Of these, 43 (74.2%) answered “yes” (n = 23) or “partly” (n = 20) to the question: “Are you practicing any form of time-restricted eating at the moment”? Most practiced 16:8 (n = 18) or 14:10 (n = 15) with flexibility during weekends and holidays. Six participants practiced 16:8 TRE, as in the intervention, and four practiced other forms of TRE. Of those not continuing with TRE, or only partly continuing with TRE, reasons are given in Table 5.

The mean IBS-SSS scores for this subgroup (n = 58) answering the follow-up survey was 287.8 (SD 85.5) at baseline, reduced significantly to 178.4 (SD 102.6; *p* < 0.001) after the intervention. At 12 months follow-up the mean IBS-SSS score was 207.3 (SD 103.5), and even though the score was higher than after the intervention, there still was a significant reduction from baseline (*p* < 0.001).

Regarding self-reported physical health, mean scores in this subgroup (n = 58) increased significantly from 6.07 (SD 1.59) at baseline to 6.69 (SD 1.72; *p* = 0.003) after the intervention. At 12-month follow-up the self-reported physical health score was 6.60 (SD 1.65), which still was a significant increase from baseline (*p* = 0.036). For self-reported mental health, the scores in this subgroup (n = 58) were 6.53 (SD 2.13) at baseline increased significantly to 6.98 (SD 1.90; *p* = 0.031) after the intervention. At 12 months follow-up the self-reported mental health score was 6.93 (SD 1.87), not a significant increase from baseline, however a trend (*p* = 0.069).

In the follow-up questionnaire, 43 participants provided open-ended comments regarding perceived health effects beyond IBS symptom changes. These responses represent descriptive participant feedback rather than a formal qualitative analysis. Frequently mentioned experiences included improved sleep (37%), better weight regulation (30%), reductions in general bodily discomfort (19%), improved mood (16%), increased energy (16%), and fewer food cravings (12%).

## 4. Discussion

To our knowledge, this pilot study is the first to evaluate the potential effects of TRE as a potential adjunct IBS treatment. Using the validated IBS-SSS questionnaire, we observed reductions in symptom severity, along with improvements in self-reported physical and mental health. Notably, patients with IBS-C showed the largest decrease in IBS-SSS scores. Post hoc effect size estimation (Cohen’s d = 0.89) suggests a large within-group change; however, this should be interpreted cautiously given the uncontrolled pilot design.

### 4.1. Study Design and Feasibility

We conducted a single-group, pre–post intervention pilot study, in which participants with IBS underwent an 8-week time-restricted eating regimen. Outcomes were assessed at baseline and after the intervention to explore their potential efficacy. Participants were allowed to select either early or late TRE while maintaining a 16 h fasting window—a flexible approach that was meant to facilitate high retention. The retention rate at the end of the intervention also suggests that the dietary protocol was feasible for most participants, indicating that this meal timing approach may be useful for a broader population of individuals with IBS. Additionally, the participants reported that the Facebook group created a sense of community and allowed them to share experiences and support each other throughout the study. This social support may have contributed to the high level of study completion, as shown by Kestyüs and colleagues [32]. The absence of dietary restrictions and financial costs further supports its potential as a sustainable lifestyle intervention. The single-group design without a control arm limits causal inference, and the duration restricts conclusions to short-term effects. However, the exploratory 12-month follow-up suggested possible maintenance of symptom improvement among respondents.

### 4.2. Comparison with Existing Literature

As there are currently no published studies investigating the use of TRE in IBS populations, direct comparisons with existing studies are not possible. The only study that has examined the effects of fasting on IBS symptoms was conducted by Kanazawa et al. In this study, the participants underwent a 10-day water fast, followed by 5 days of refeeding. The study demonstrated a reduction in certain symptoms in patients with IBS-D, however with a more extreme form of fasting than TRE [33]. As this trial evaluated effects via the Bristol stool scale, and not the IBS-SSS, a direct comparison is not feasible.

### 4.3. Comparison with Dietary Approaches—IBS Symptoms

We observed a mean reduction in IBS-SSS score of −100.2, which is comparable to reductions reported in studies evaluating dietary interventions such as the low-FODMAP diet, gluten-free diets, low-carbohydrate dietary approaches, as well as diet combined with standard dietary advice [31]. A 2024 systematic review and meta-analysis examined 23 studies comparing low-FODMAP diets and gluten-free diets to standard diets in patients with IBS [34]. Included studies varied in duration (3–12 weeks), and some focused exclusively on the IBS-D population. In studies comparing a low FODMAP diet with standard dietary advice, the low FODMAP diet was associated with greater reductions in IBS symptom severity, as measured by the IBS-SSS [34]. In contrast, comparisons between gluten-free diets and standard diets did not show statistically significant changes in IBS-SSS scores [34]. The ability of a very low carbohydrate diet to reduce symptoms in patients with IBS-D [16] could be attributed to the exclusion of certain FODMAPs; however, it is also possible that the anti-inflammatory effect of ketone bodies contributes to symptom improvement [26,28]. Although our pilot study cannot be directly compared to any of these randomized controlled trials; the finding that reductions in symptoms were comparable to more established treatment options suggest that further investigation into TRE as a potential treatment for IBS is warranted.

### 4.4. Comparison with Dietary Approaches—Quality of Life

Existing studies have compared dietary interventions in relation to quality of life using the Irritable Bowel Syndrome Quality of Life Instrument (IBS-QOL), and improvements have been reported from both from low FODMAP diets and Mediterranean diets [34]. Notably, Mediterranean diets demonstrate a greater improvement in quality of life than more restrictive approaches, including low FODMAP diets, gluten-free diets, low-carbohydrate diets, and a combination of low FODMAP diets with fiber supplementation [34]. This finding is somewhat unexpected, as Mediterranean diets do not impose restrictions based on gastrointestinal symptom triggers [35], contrasting other therapeutic dietary approaches. Instead, Mediterranean diets are characterized by high overall dietary quality and greater flexibility, suggesting that a less restrictive dietary approach may contribute positively to quality of life [35]. Flexibility in food choice is also a defining characteristic of time-restricted eating, which does not restrict specific food types and may therefore support quality of life.

Although a small reduction in body weight was observed in a subset of participants, it is unclear whether changes in BMI contributed meaningfully to symptom improvement. Evidence on the relationship between weight loss and symptom severity in individuals with IBS is limited. A prospective cohort study in individuals with severe obesity suggested that substantial weight reduction may be associated with improvements in IBS symptoms [36]. However, the participants in that study had markedly higher baseline BMI compared with the present study, limiting the generalizability of those findings to populations with normal weight or moderate overweight. Furthermore, the symptom improvement observed among individuals who lost weight may reflect qualitative changes in diet during the weight-loss phase rather than weight reduction per se. It remains unclear whether participants in our study who lost weight simply reduced portion sizes of their habitual foods or whether they made more substantial alterations to their dietary composition. Improvements in dietary quality—such as increased intake of whole and minimally processed foods, combined with reduced consumption of ultra-processed products—may influence gastrointestinal symptoms [19,37]. Accordingly, changes in diet quality could represent a significant confounding factor when interpreting the relationship between BMI reduction and symptom improvement in IBS. Given that the study did not aim to evaluate the effects of weight change, the observed BMI shifts should be interpreted cautiously and considered an exploratory finding.

An important consideration when interpreting our findings relates to the timing of the eating window. Previous studies have suggested that early time-restricted eating (eTRE) may confer greater metabolic benefits than late time-restricted eating (lTRE), potentially due to closer alignment with circadian rhythms [38,39]; however, the evidence is still inconclusive. The primary objective of the present study was to examine whether time-restricted eating, as a general behavioral strategy, could be beneficial irrespective of the timing of the eating window. Accordingly, participants were allowed to self-select either an early or late eating window and to alternate between these approaches as needed to facilitate integration into daily life. If implemented in clinical practice, this flexible approach to time-restricted eating may represent a low-cost and pragmatic strategy that can be adapted to individual preferences and life circumstances. In the present study, a 16:8 protocol was applied; however, it is plausible that less restrictive regimens (e.g., 15:9 or 14:10) could also be beneficial for individuals with IBS, provided that they extend the habitual fasting duration.

### 4.5. Individual Differences

Although most participants experienced improvements in symptoms, a subset reported symptom worsening. This finding underscores substantial interindividual variability in response to time-restricted eating, suggesting that underlying biological or behavioral factors may modulate the efficacy of this intervention in patients with IBS. This study did not collect detailed individual dietary intake or behavioral data during the eating window, nor was it designed to evaluate correlations between symptom change, individual weight trajectories, or timing of eating windows. Because participants were allowed to switch between early and late schedules, meaningful classification by timing was not possible. Future studies should incorporate more detailed behavioral and dietary monitoring to enable correlation analyses and identify predictors of response. Such heterogeneity highlights the need for more personalized approaches to IBS management and reinforces the importance of expanding the therapeutic toolbox available to clinicians.

### 4.6. Self-Reported Physical and Mental Health

Given the well-established burden from comorbidities, improvements in gastrointestinal symptoms may be accompanied by changes in physical and mental well-being. In this study, modest but statistically significant improvements were observed in self-reported physical and mental health following the 8-week intervention. Although these measures were exploratory and based on non-validated scales, the findings suggest that future studies should consider including validated instruments to more comprehensively assess changes in physical and mental health. Despite considerable inter-individual variability, the qualitative findings from open-ended questions in the follow-up survey suggest that TRE may confer benefits for co-morbid symptoms frequently reported in IBS. These observations should be interpreted strictly as hypothesis-generating, given that the follow-up was exploratory, not pre-specified, and relied on self-reported qualitative data.

### 4.7. Potential Mechanisms

Biological pathways that may mediate the observed effects include fasting-induced modulation of the migrating motor complex [22], metabolic switching with ketogenesis and activation of autophagy [21,23,25], modulation of the gut microbiota [24] and broader cellular and tissue-level responses [19,26,27,28]. While these mechanisms are biologically plausible—particularly given their overlap with pathophysiological processes implicated in IBS, such as impaired motility, low-grade inflammation, epithelial barrier dysfunction, and dysbiosis—this pilot study was not designed to investigate mechanistic pathways. Accordingly, any proposed explanations should be interpreted with caution. Nevertheless, the alignment between fasting-induced biological responses and established IBS-related mechanisms highlights the need for future mechanistic studies to directly examine how time-restricted eating may influence gastrointestinal function and symptom generation in IBS.

### 4.8. Clinical Implications and Future Research

Our findings suggest that time-restricted eating may represent a simple, cost-free, and feasible approach to IBS symptom management. Its inherent flexibility may facilitate integration into a wide range of everyday life contexts, potentially empowering patients to take an active role in managing their gastrointestinal symptoms.

However, to establish the efficacy, sustainability, and underlying mechanisms of time-restricted eating in IBS, future studies should employ more rigorous designs, including randomized controlled trials with longer follow-up periods. Such studies should incorporate validated measures of quality of life and mental and physical health, objective biological markers (e.g., high-sensitivity C-reactive protein (hs-CRP) and fecal calprotectin), and gut microbiota profiling. Objective assessments of dietary adherence, potentially including continuous glucose monitoring, should also be considered. In addition, future research should evaluate whether habitual dietary patterns are maintained over time and examine the applicability of time-restricted eating in populations excluded from the present pilot study, including individuals with conditions that produce IBS-like symptoms.

### 4.9. Limitations

Several limitations must be acknowledged. Our sample was predominantly female and recruited through the Norwegian Gastrointestinal Association and social media, introducing potential sex and selection bias. The participants were self-selected and highly motivated, which may have contributed to positive expectancy effects. Symptom assessment relied on self-reporting, raising concerns about subjectivity, placebo and Hawthorne effects and unmeasured confounding factors such as concurrent dietary changes. Compliance was not objectively monitored, and changes in BMI may have influenced outcomes. As this is the first study to investigate the potential effect of TRE on IBS symptoms, it was not possible to perform an a priori power calculation. Although participant recruitment was relatively successful, the lack of power calculations introduces uncertainty regarding the reliability and generalizability of the finding. The single-group pre–post design without a control group precludes causal inference, and improvements may partially reflect regression to the mean, expectancy effects, or natural symptom fluctuation. Attrition during the intervention and reduced participation at long-term follow-up introduce potential selection bias. IBS diagnosis was self-reported and not independently verified, raising the possibility of misclassification. The study was retrospectively registered after completion of data collection, which represents a methodological limitation related to transparency and should be considered when interpreting the findings. Finally, subgroup analyses were exploratory and not adjusted for multiple comparisons. These findings should therefore be interpreted as preliminary and hypothesis-generating.

## 5. Conclusions

This exploratory pilot study suggests that time-restricted eating may be associated with improvements in IBS symptom severity. However, the uncontrolled design and reliance on self-reported outcomes preclude causal inference. These findings should therefore be considered preliminary and hypothesis-generating, and randomized controlled trials are needed to determine whether TRE has a true therapeutic effect in IBS.

## Figures and Tables

**Table 1:** Baseline characteristics of participants (n = 97).

Women (frequency, %)	90 (92.8%)
Age, years (mean, SD)	42.5 (12.9)
Baseline BMI (kg/m^2^)	26.2 kg/m^2^
IBS subtype (Rome IV) (frequency, %)	
Constipation	29 (29.9%)
Diarrhea	21 (21.7%)
Mixed	41 (42.3%)
Unclassified	6 (6.2%)

**Table 2:** IBS symptom severity score (IBS-SSS) before and after the intervention (n = 97).

	Before Intervention	After Intervention	Difference	*p*-Value ^1^
	Mean (SD), [CI]	Mean (SD), [CI]	Mean (SD), [CI]	
IBS-SSS score (range 0–500)	274.5 (89.3) [256.5, 292.5]	174.3 (96.3) [154.9, 193.7]	−100.2 (112.3) [77.6, 122.8]	<0.001

^1^ Paired sample *t*-test, two-sided. Data are presented as the mean (SD), unless otherwise specified.

**Table 3:** IBS symptom severity scale (IBS-SSS) scores for IBS-C, IBS-D, and IBS-M before and after the intervention, and the corresponding mean changes in scores before and after the intervention (n = 91).

IBS-SSS Score Subgroup Analysis (Range 0–500)	Before Intervention	After Intervention	Difference	*p*-Value ^1^
	Mean (SD), [CI]	Mean (SD), [CI]	Mean (SD), [CI]	
Constipation	285.6 (95.2) [249.3, 321.8]	160.4 (84.4) [128.3, 192.4]	−125.2 (117.9) [80.4, 170.1]	<0.001
Diarrhea	245.3 (96.9) [201.2, 289.4]	169.3 (105.6) [121.3, 217.4]	−76.0 (100.4) [30.3, 121.7]	<0.005
Mixed	280.1 (78.9) [255.2, 305.0]	186.0 (103.0) [154.5, 219.4]	−93.1 (107.4) [59.2, 127.0]	<0.001

^1^ Paired sample *t*-test, two-sided. Data are presented as mean (SD).

**Table 4:** IBS-SSS severity categories based on score. Participants were categorized into remission, mild, moderate, or severe scores, corresponding to <75, 75–175, 175–300, and >300, respectively (n = 97).

IBS-SSS Categories (Score)	Before Intervention (Frequency, %)	After Intervention (Frequency, %)
Remission (<75)	0	12 (12.4%)
Mild severity (75–175)	15 (15.5%)	39 (40.2%)
Moderate severity (175–300)	44 (45.4%)	36 (37.1%)
Severe severity (>300)	38 (39.2%)	10 (10.3%)

**Table 5:** Reasons for not continuing with TRE (n = 58).

	Frequency (%)
Did not work on my IBS symptoms	7 (12.1)
My IBS symptoms got worse	0 (0)
Did not work for my life situation (work, moving, children, other disease, etc.)	5 (8.6)
I got strong physical discomfort (nausea, hunger, etc.)	3 (5.2)
Other	4 (7.0)
Total	19 (32.8)

## Data Availability

Due to ethical and privacy considerations, the data are not publicly available. De-identified data may be made available from the corresponding author upon reasonable request.

## Abbreviations

The following abbreviations are used in this manuscript:

BMI	Body mass index
hs-CRP	High-sensitive C-reactive protein
FMT	Fecal microbiota transplantation
FODMAP	Fermentable Oligo-, Di-, Monosaccharides and Polyols
IBS	Irritable bowel syndrome
IBS-SSS	Irritable bowel syndrome symptom severity scale
IBS-C	Irritable bowel syndrome (Constipation)
IBS-D	Irritable bowel syndrome (Diarrhea)
IBS-M	Irritable bowel syndrome (Mixed)
IBS-U	Irritable bowel syndrome (Unclassified)
REK	Regional Committee for Medical and Health Research Ethics
TRE	Time-restricted eating
eTRE	Early time-restricted eating
lTRE	Late time-restricted eating
